# Single‐Cell Profiling Identifies Skin‐Resident Memory CD4^+^ T Cells as a Potential Correlate of Immunity During *Staphylococcus aureus* Skin Infection

**DOI:** 10.1002/eji.70268

**Published:** 2026-08-24

**Authors:** Jonah Clegg, Giovanni Cova, Alberto Carignano, Megan Smith, Emiliano Chiarot, Simona Tavarini, Chiara Sammicheli, Nicholas Rachmaninoff, Serena Vastola, Silvia Guidotti, Emilio Siena, Monia Bardelli, Fabio Bagnoli, Michela Brazzoli, Rachel M. McLoughlin, Elisabetta Soldaini

**Affiliations:** ^1^ GSK via Fiorentina 1 Siena Italy; ^2^ Host Pathogen Interactions Group, School of Biochemistry and Immunology Trinity Biomedical Sciences Institute, Trinity College Dublin Dublin 2 Ireland; ^3^ INGM, C/O Fondazione IRCCS Cà Granda Ospedale Maggiore Policlinico di Milano Via Francesco Sforza Milano Italy; ^4^ GSK Rockville Maryland USA

**Keywords:** *S. aureus*, scRNA‐seq, skin infection, tissue‐resident memory T cells, γδ T cells

## Abstract

*Staphylococcus aureus* is a leading cause of skin and soft tissue infections (SSTIs), yet the cellular mediators of protective memory remain incompletely defined. Using a self‐resolving murine SSTI model, we combined longitudinal single‐cell RNA sequencing with flow cytometry to characterize immune memory within the skin following infection. We identified a persistent population of CD4^+^ T cells that remained in the skin after bacterial clearance and acquired a transcriptional program consistent with tissue residency, including expression of genes associated with tissue retention and long‐term residence. Phenotypic analysis confirmed the emergence of a skin‐resident memory T cell (Trm) population that persisted well beyond resolution of infection and displayed characteristics of a clonal T cell response. Upon reinfection, protective immunity was associated with rapid recall responses from these resident cells and was maintained despite FTY720‐mediated blockade of lymphocyte egress from secondary lymphoid organs, demonstrating that circulating lymphocytes were dispensable for protection. Together, these findings identify infection‐induced CD4^+^ Trms as a durable component of immune memory following *S. aureus* SSTI and support a role for tissue‐resident immunity in protection against recurrent infection, highlighting CD4^+^ Trms as a potential target for future vaccine strategies.

## Graphical abstract

*Staphylococcus aureus* skin infection generates long‐lived tissue‐resident memory T cells that provide enhanced local recall responses to reinfection, leading to improved bacterial clearance. Skin Trm could therefore be a potential correlate of protective immunity and targeted in future vaccine development.

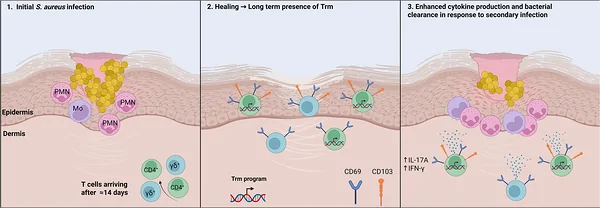

**Abbreviations**
CFU: colony‐forming units
DEGs: differentially expressed genes
EM: effector memory
FACS: fluorescence‐activated cell sorter
FTY720: Fingolimod
HK: heat‐killed
IFN‐γ: interferon‐gamma
Inf: infected
p.i.: postinfection
PCA: principal component analysis
*S. aureus*
: *Staphylococcus aureus*
scRNA‐seq: single‐cell RNA‐sequencing
SSTIs: Skin and Soft Tissue Infections
Tcm: central memory T cell
Tcon: Conventional T cell—non‐Treg αβ T cell
Tem: effector memory T cell
Tn: naïve T cell
Treg: regulatory T cell
Trm: tissue‐resident memory T cell
UMAP: uniform manifold approximation and projection

## 1 Introduction

Despite widespread exposure and frequent colonization, protective immunity against *Staphylococcus aureus* at barrier sites is often incomplete, resulting in recurrent infection and progression to invasive disease. Among the clinical manifestations of *S. aureus* infection, skin and soft tissue infections (SSTIs) are the most prevalent [1, 2]. In the United States, *S. aureus*‐associated SSTIs cost $4.5 billion in annual healthcare expenditure [3]. Although antibiotic therapy remains the cornerstone of treatment, *S. aureus* displays a remarkable ability to acquire resistance across all major classes of antimicrobials. These features underscore the need to define immune mechanisms capable of mediating durable protection against *S. aureus* SSTIs.

Evidence from both human disease and experimental models has implicated CD4^+^ T cells as central mediators of protective immunity to *S. aureus*, with particular relevance at barrier tissues. Individuals with congenital or acquired impairments in CD4^+^ T cell number or function show a disproportionate susceptibility to mucocutaneous infections, including SSTIs, relative to immunocompetent individuals [4, 5]. In support of this, patients with active HIV infection demonstrate a 16.5‐fold increased risk of developing *S. aureus* bacteremia, while the relative risk for *S. aureus* SSTI increases to approximately 60‐fold [5, 6]. Similarly, individuals with Hyper‐IgE Syndrome, resulting from mutations in the transcription factor STAT3 that impair Th17 differentiation and function, display heightened susceptibility to *S. aureus* infections that are largely restricted to epithelial barrier sites such as the skin and lungs [4]. These clinical observations are supported by mechanistic studies in murine models of *S. aureus* infection and vaccination. Adoptive transfer of CD4^+^ T cells from mice previously challenged with *S. aureus* SSTI conferred protective immunity to naïve recipient animals [7]. In a model of systemic *S. aureus* infection followed by secondary cutaneous challenge, depletion of CD4^+^ T cells abrogated the development of protective immunity against subsequent SSTI [8]. Furthermore, in vaccination studies demonstrating protection against both *S. aureus* bacteremia and SSTI, B cell deficiency eliminated vaccine‐mediated protection from bacteremia, whereas protection in two independent SSTI models was preserved [9]. Consistent with a central role for CD4^+^ T cells in cutaneous protection, previous work has identified the T cell–derived cytokines IL‐17 and, to a lesser extent, IFN‐γ, as essential mediators of immunity during S. aureus SSTI [7, 10].

To date, studies investigating the protective function of CD4^+^ T cells during *S. aureus* SSTI have exclusively focused on the circulating CD4^+^ T cell response, neglecting a potential role for CD4^+^ tissue‐resident memory T cells (Trms). Trms represent a subset of memory T cells defined by their disequilibrium with the circulatory immune system [11]. In recent years, nonredundant protective roles for CD4^+^ Trms have been described in various infection settings [12, 13, 14, 15, 16, 17], with murine models identifying skin‐resident CD4^+^ T cells as key mediators of protective immunity against *C. albicans* and *L. major* [18, 19, 20]. Infection with *C. albicans* was shown to drive the infiltration in the skin of CD4^+^ T cells that, upon resolution, adopted a tissue‐residency phenotype, which produced large amounts of IL‐17 and mediated protection during secondary infection [18]. A similar finding was reported during infection with *L. major*, whereby infection drove the development of skin‐resident CD4^+^ Trms of a largely Th1 phenotype that contributed to enhanced protection during reinfection [19, 20]. In addition, in a murine model of *S. aureus* pneumonia, induction of Th1‐type Trms residing in lungs has been shown to be protective [21]. Taken together, these studies form the basis for the hypothesis that skin‐resident CD4^+^ T cells may prove protective during recurrent SSTI with *S. aureus* [22].

Given its dominance as an asymptomatic colonizer of multiple bodily surfaces, healthy humans harbor pre‐existing *S. aureus*‐specific CD4^+^ T cells [23]. Accordingly, previous work from the Soldaini group has identified a CD4^+^ Trm population in human skin from healthy subjects that proliferates and produces IL‐17 and, to a lesser extent, IFN‐γ upon stimulation with heat‐killed *S. aureus* [23]. However, whether such cells contribute to protection against *S. aureus* SSTI is as of yet not known. As such, in this study, the formation and phenotype of Trms during primary *S. aureus* infection was investigated using a mouse model of SSTI combined with a systems immunology approach to map immune cell dynamics during *S. aureus* skin infection. This revealed a population of skin‐infiltrating CD4^+^ T cells that persisted after infection resolution and adopted a tissue‐resident transcriptional profile. These CD4^+^ Trm cells produced IL‐17 and IFN‐γ in response to re‐exposure to *S. aureus*. Blocking lymphocyte infiltration during reinfection did not impair immunity, compatible with protection induced by prior exposure being mediated by resident, not circulating, T cells. These findings identify infection‐induced CD4^+^ Trms as a potential target for future *S. aureus* vaccine strategies.

## 2 Materials and Methods

### 2.1 Murine Model of *S. aureus* Skin Infection

*S. aureus* USA300 LAC strain was used throughout the study. Bacteria were grown overnight, diluted, and regrown for 3 h to reach the early exponential phase (OD600 = 2). Bacteria were centrifuged, washed, and re‐suspended in cold phosphate‐buffered saline (PBS) at a concentration of 2 × 10^6^ Colony Forming Units (CFU)/50 µL. Six‐week‐old C57BL/6N female mice were anesthetized with isoflurane (induction with 4–5% for 1 min, maintenance with 2.5% up to 5 min) and shaved the day before infection. Animals were shaved using an electric razor followed by a commercially available cream applied on the partially shaved skin. Incubation time was up to 3 min, then the cream was removed by washing with plenty of warm water. The day after, animals were anesthetized under the same conditions using isoflurane and infected subcutaneously on the right flank with 50 µL of *S. aureus* (2 × 10^6^ CFU) or PBS as a negative control. Identical conditions were used for reinfection experiments 42 days after the primary infection. In this case, animals were challenged with an identical dose of *S. aureus* at the site of the healed initial infection.

Clinical signs, including bodyweight, severity of skin lesions, and mouse motility, were monitored daily for up to 14 days after infection or until animals were sacrificed. To quantify bacterial load (CFU), infected skin tissue was harvested and homogenized using a gentleMACS Octo Dissociator (Miltenyi Biotec). Homogenized tissue was filtered through a 70 µm filter, serially diluted, and plated on Tryptic Soy Agar (TSA) plates supplemented with 10% sheep's blood for enumeration. The development of dermonecrosis was assessed at various time points postinfection by photographing sedated mice with a 5 cm scale block. Anesthesia was performed using the same conditions used for shaving and infection. Lesion size was calculated using Fiji ImageJ software.

### 2.2 Preparation of Single Cell Suspensions

Infected skin (approximately 200 mg) was harvested, weighed, and minced using sterile scissors and forceps. Skin was enzymatically digested for 45 min in cRPMI containing 2 mg/mL Collagenase XI (Sigma #C9407), 0.5 mg/mL Hyaluronidase (Sigma #H3506), and 0.1 mg/mL DNase (Sigma #11284932001). For cell sorting intended for scRNA‐seq, 200 µg/mL Gentamycin (Sigma #G3632) and 40 µg/mL Vancomycin (Sigma #V2002) were added during the enzymatic digestion to kill extracellular *S. aureus*. The resulting suspensions were filtered through 70 and 40 µm cell strainers before centrifugation and resuspension for flow cytometry. Spleen samples were processed by crushing through a 70 µm cell strainer, followed by centrifugation, red blood cell lysis, and washing prior to counting and plating.

### 2.3 Phenotypic Characterization by Flow Cytometry

Single‐cell suspensions were stained with a viability dye (Live/Dead Near‐IR, ThermoFisher #L10119), blocked with anti‐mouse CD16/CD32 (BD #553141), and stained extracellularly for 20 min at 4°C. Cells were stained for CD3 (PerCP‐Cy5.5 BD #561108), CD4 (BUV737; BD #612844), CD8 (BUV805; BD #612898 and RB780; BD #568692), CD19 (APC; BD #550992), γδ TCR (BV711; BD #563994), CD44 (PE‐Cy5; BD #553135 and BUV496; BD #741057), CD45 (BUV395; BD #564279 and FITC; BioLegend #147710), CD62L (BV605; BD #563252 and APC; BD #553152), CD69 (BV480; BD #746813 and BUV661; BD #741478), CD103 (PE‐CF594; BD #569678), and Ly6G (BV786; BD #740953). Cells were then fixed with Cytofix (BD # 554655) for 15 min.

### 2.4 Intracellular Cytokine Staining (ICS)

For ex vivo analysis of T cell effector function postinfection, cells were plated in cRPMI supplemented with anti‐CD28 and gentamicin for a total of 4 h. Brefeldin A was added for the final 2 h. Cells were stained extracellularly and then permeabilized using Cytofix/Cytoperm (BD #554714), followed by intracellular staining for IFN‐γ (PE‐Cy7; BD #557649 and BV480; BD #566097) and IL‐17 (BV421; BD #563354) for 30 min at RT. All flow samples were read using a BD FACS Fortessa or BD FACS Symphony A5. Data were analyzed using FlowJo software version 10.10.0 (BD).

### 2.5 Single Cell RNA Sequencing (scRNA‐Seq) and T Cell Receptor Sequencing (TCR‐seq)

Cells were harvested from infected skin tissue at timepoints reflecting homeostasis (Day 0), early innate responses (Days 1 and 3), adaptive responses (Day 13), and skin‐resident memory (Day 42), as well as 24 h post‐rechallenge. Cells were stained extracellularly with CD45, Ly6G, and TotalSeq cell hashing antibodies. Live, CD45^+^Ly6G^−^ cells were sorted using a BD FACSAria Fusion (4000 cells per sample, except Day 0, which ranged from 1500 to 4000 cells).

Sc‐RNA‐seq and TCR‐seq were performed using the Chromium Gel Bead‐in‐Emulsion Single Cell 5’ Reagent Kit and V(D)J Reagent Kit, respectively (10x Genomics). Sorted cells were encapsulated with gel beads containing barcode nucleotides and unique molecular identifiers to enable single‐cell identification of cDNA libraries. Following reverse transcription and cDNA amplification, libraries were constructed, fragmented, and ligated with adapter sequences for sequencing. Sequencing was performed on an Illumina NovaSeq 6000 instrument using the NovaSeq 6000 S1 Reagent Kit v1.5 (Illumina #20028318) following manufacturer's instructions.

### 2.6 Data Analysis

The resulting 10X data were analyzed using the Scanpy package in Python. Quality control (QC) excluded doublets (Scrublet threshold 0.25), cells expressing fewer than 1000 genes, genes appearing in fewer than three cells, cells with over 3500 total counts, and cells with mitochondrial gene percentages exceeding 3.5%. Counts were normalized to 10,000 and logarithm‐transformed for Principal Component Analysis (PCA) and Uniform Manifold Approximation and Projection (UMAP). Leiden clustering was employed (resolution 0.65) to identify 14 immune cell clusters. Cell phenotyping relied on comparing the top 20 Differentially Expressed Genes (DEGs) of each cluster against gene lists from the Immunological Genome Project (immgen.org). Simpson and Shannon indices of TCRs sequenced across skin samples taken at different times (0–42) were calculated by using the mean clonality metrics from 1000 jackknife subsamples of 40 cells to account for differences in total number of T cells. Differences in clonality were assessed using nonparametric tests. Since this study is cross‐sectional (i.e., different mice were sacrificed at different time‐points), it was not possible to track individual clones over time. To allow for inference of potential specificity overlap, TCR‐seq data were analyzed using TCRdist3, an open‐source Python package, which allowed to cluster TCRs based on similarity and made cross‐timepoint/sample TCR comparisons. TCRdist3 meta‐clonotypes (a fuzzy clustering where TCRs can fall into several meta‐clonotypes) were then grouped into separate clusters by building a network where each node represented a TCR and edges denoted whether the TCR's were contained in a shared meta‐clonotype. The igraph R package was then used to extract the connected components of the graph, which represented the final TCR clustering.

### 2.7 FTY720 Treatment

To prevent lymphocyte egress from secondary lymphoid organs during reinfection, convalescent mice were treated with FTY720 (Sigma #SML0700). Mice were injected intraperitoneally with 200 µL of FTY720 at a concentration of 90 µg/mL (or PBS control) 24 h before infection. On the day of infection, FTY720‐treated mice's drinking water was replaced with water supplemented with FTY720 such that mice drank approximately 0.3 mg FTY720/kg/day. This was calculated from internal observations that mice drank roughly 7 mL water per day.

### 2.8 Statistical Analysis

Statistical analysis utilized GraphPad Prism version 8 or the Python package Scanpy for transcriptomic analyses. Normality testing was applied. Comparisons between two groups utilized *t*‐tests. Comparisons involving three or more groups across a single timepoint used a one‐way ANOVA with an appropriate posttest (e.g., Dunnett's test). Analyses involving three or more groups across multiple conditions used a two‐way ANOVA with an appropriate posttest. A *p*‐value <0.05 was considered statistically significant.

### 2.9 Ethics Statement

Animal husbandry and experimental procedures were ethically reviewed and carried out in accordance with European Directive 2010/63/EU, Italian Decree 26/2014, and GSK Vaccines’ Policy on the Care, Welfare and Treatment of Animals, and were approved by the Italian Ministry of Health (authorization n° 669/2022‐PR). Animals were kept in an AAALAC‐accredited facility. Briefly, animals were randomly distributed in different experimental groups in individually ventilated cages (IVC). At the end of an acclimation period of at least 5 days, each animal was identified by an individual tattoo on the tail. The animal room conditions were the following: temperature 21°C (±3°C), relative humidity 50% (range 30%–70%), and 12 h/12 h light/dark cycle. Pressure, temperature, and relative humidity were recorded continuously by room probes, while the IVC system recorded the individual motors’ performance. The light cycle setting was ensured by a validated alarm system. Female C57BL/6N Specific Pathogen Free (SPF) and C57BL/6J Specific and Opportunistic Pathogen Free (SOPF) mice were sourced from Charles River Laboratories (Germany) and housed in a GSK Vaccines animal housing unit. SOPF mice that are *S. aureus*‐free were used for the majority of the experiments, including the scRNA‐seq, to avoid confounding effects due to uncontrolled natural exposure to *S. aureus*. SOPF and SPF mice behaved similarly for the readouts of the reported experiments.

## 3 Results

### 3.1 Characterization of the Immune Response to *S. aureus* Skin Infection via Single‐Cell RNA‐Sequencing Reveals Multiple Populations of Immune Cells Present Within Infected Skin

A murine model of self‐resolving *S. aureus* skin infection was established using female C57BL/6 mice infected with the USA300 LAC strain (Figure S1A). Bacterial burdens in the skin declined over time and were largely cleared by day 15 (Figure S1B), consistent with previous reports [10, 24]. As expected, neutrophils (CD45^+^Ly6G^+^) represented the dominant immune population present in the skin during acute infection, peaking at day 1 when they comprised ∼80% of total skin leukocytes, before declining alongside bacterial clearance (Figure S1C,D). The close correlation between neutrophil infiltration and bacterial survival highlights their role as key innate antimicrobial effectors. Given neutrophils’ numerical dominance, leukocytes that are not neutrophils (i.e., CD45^+^Ly6G^−^ cells) were FACS sorted from skin cell suspensions prepared from infected mice for downstream scRNA‐seq analysis.

**FIGURE 1:**
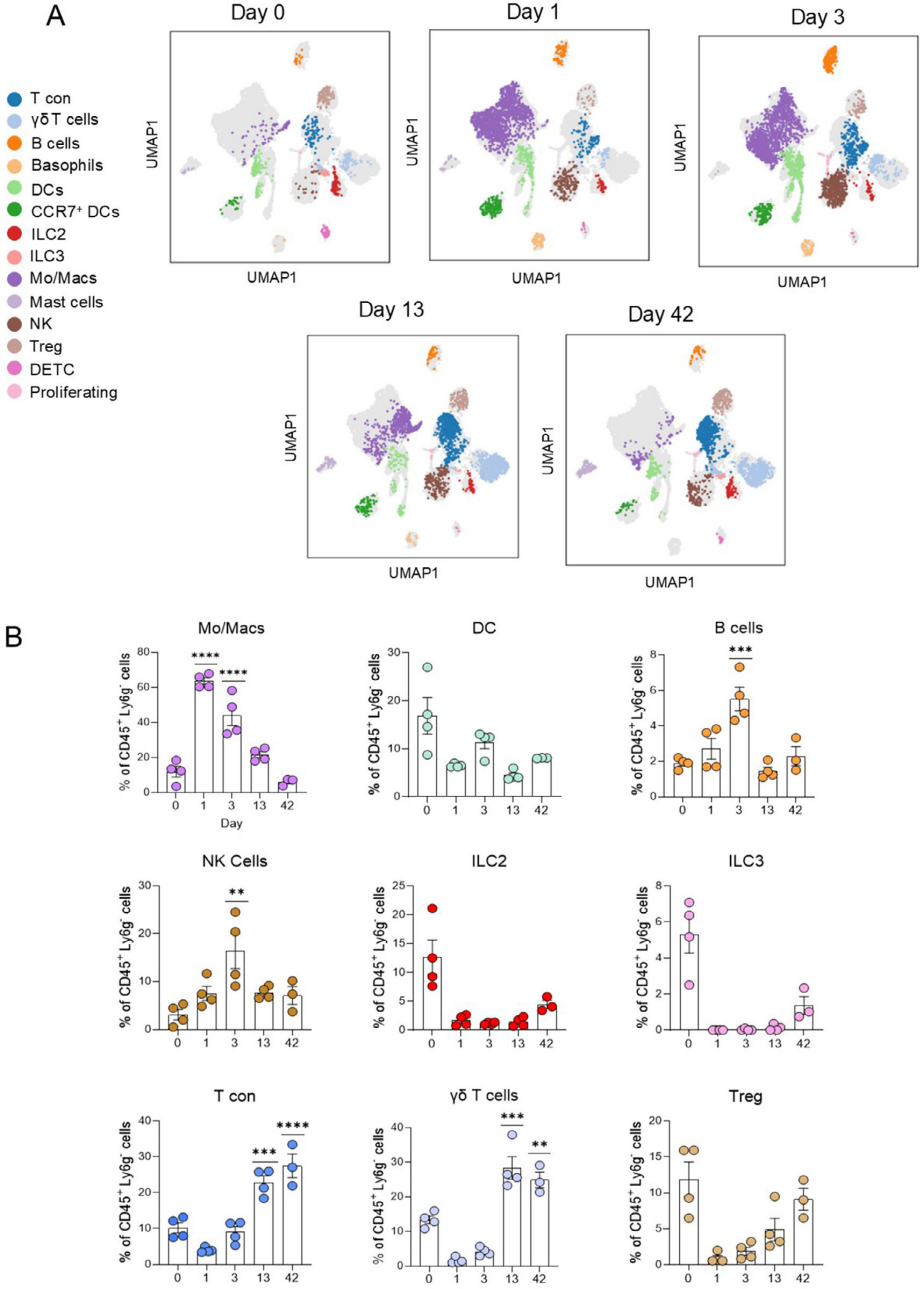
*S. aureus* skin infection drives recruitment of multiple immune cell populations that follow distinct kinetic profiles. (A) C57BL/6 female mice were infected subcutaneously with 2 × 10^7^ CFU of *S. aureus* USA300 LAC strain. At days 0 (non infected), 1, 3, 13, and 42 post infection (p.i.), skin was harvested, a single‐cell suspension was prepared, and stained. Live, single non‐neutrophil leukocytes (CD45^+^ Ly6G^−^) cells were FACS‐sorted, scRNA‐seq was performed, and data were analyzed. UMAP projections with all immune cell subsets from all mice sacrificed at each time point are shown (*N* = 4 mice/time point). (B) The percentages of exemplary populations as a proportion of total sequenced CD45^+^Ly6G^−^ cells are shown (*N* = 4 mice/time point). Statistical analysis was performed comparing each time point against day 0 using one‐way ANOVA with Dunnett's test for multiple comparisons. **p ≤ 0.01. ***p ≤ 0.001. ****p ≤ 0.0001.

Time points reflecting homeostasis (Day 0, no infection), early innate responses (Days 1 and 3 postinfection, p.i.), adaptive responses (Day 13 p.i.), and skin‐resident memory (Day 42 p.i.) were chosen to characterize immune dynamics during *S. aureus* skin infection. ScRNA‐seq data from 4 mice at each time point were pooled and visualized in two dimensions by Uniform Manifold Approximation and Projection (UMAP) after Leiden clustering of cells with similar gene expression profiles (Figure 1A). Cell phenotyping was performed using the top 20 differentially expressed genes (DEGs) per cluster, compared against gene lists from the Immunological Genome Project (immgen.org). Conventional T cells (Tcon, non‐Treg αβ T cells), γδ T cells, NK cells, ILC2s, Treg, and B cells showed clear spatial congregation, as did innate subsets including monocyte/macrophages, dendritic cells, and mast cells (Figure 1A; the top 5 DEGs for each subset are shown in Figure S2). To examine cellular kinetics, each time point was analyzed separately and the relative abundance of populations compared (Figure 1B). For example, monocyte/macrophages (Mo/Macs) peaked at Day 1, remained high at Day 3 and then declined; NK cells showed a transient increase at Day 3, γδ T cells and Tcon were elevated at Day 13 and remained so at Day 42, indicating adaptive memory formation in the skin (Figure 1B). Together, these data form an “immune atlas” of the nonneutrophilic immune response to *S. aureus* skin infection in mice and demonstrate the persistence of memory T cell populations in the skin after bacterial clearance and tissue repair have occurred.

### 3.2 Subset Analysis Reveals CD4^+^ T Cells of an Effector Memory Phenotype as the Dominant T Cell Population Present in the Skin Upon Resolution of *S. aureus* Infection

**FIGURE 2:**
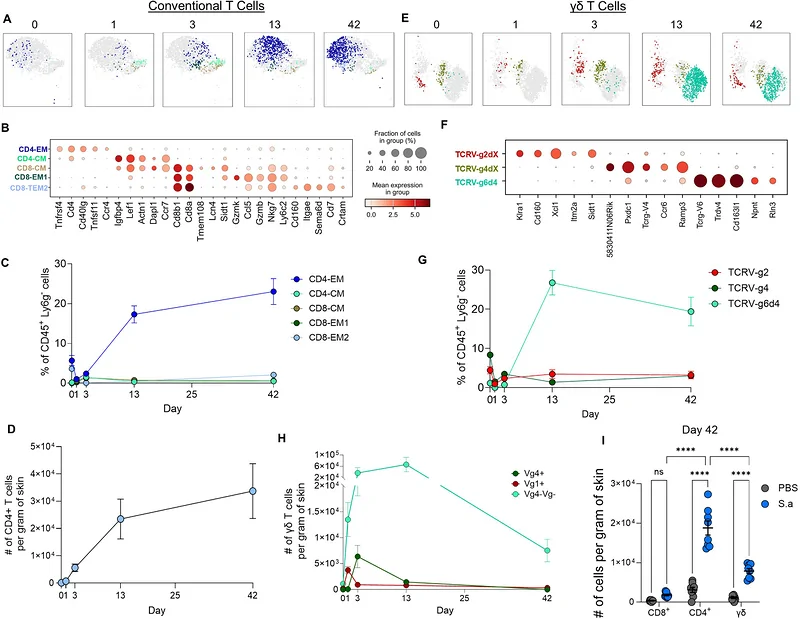
Subset analyses reveal CD4^+^ T cells of an effector memory phenotype as the dominant T cell population present in the skin upon resolution of *S. aureus* infection. C57BL/6 female mice were infected subcutaneously with 2 × 10^7^ CFU of *S. aureus* strain USA300. At days 0 (non infected), 1, 3, 13, and 42 post infection (p.i.), skin was harvested, a single‐cell suspension was prepared, and stained. Live, single non‐neutrophil leukocytes (CD45^+^ Ly6G^−^) were FACS‐sorted, scRNA‐seq was performed, and data were analyzed. Subclustering analysis was performed to reveal the differentially expressed genes (DEGs) within the overall population of conventional T cells. (B) The top 5 DEGs for each subset are reported. (A) UMAP projections showing the presence of each conventional T cell population during different phases of infection are shown. (C) The proportion of each T cell population expressed as a percentage of total sequenced CD45^+^Ly6G^−^ cells is shown (*N* = 4 for all timepoints tested except Day 42 (*N* = 3). (D) CD4^+^ T cells were quantified via FACS over the time course of infection (*N* = 4). Data displayed are a representative experiment performed 3 times. (E, F) UMAP projections showing the presence of each subset of γδ T cells throughout infection and the top 5 DEGs between each subset. (G) The proportion of each population expressed as a percentage of total sequenced CD45^+^Ly6G^−^ cells is shown (*N* = 4). (H) γδ T cell subsets were quantified via FACS during *S. aureus* infection (*N* = 4); a representative experiment of three independent experiments is shown. (I) Skin was harvested from mice 42 days p.i. or after PBS injection, and T cell subsets were quantified via FACS (*N* = 7–8, two independent experiments). Statistical analysis was performed via two‐way ANOVA. ****p ≤ 0.0001.

Following the observed expansion of Tcon cells during *S. aureus* infection, we performed in‐depth analysis of them across all time points. DEGs analysis revealed five distinct populations: two CD4^+^ and three CD8^+^ that were designated as central memory (CM) or effector memory (EM) based on high or low *Ccr7* expression, respectively (Figure 2A,B). Interestingly, the CD4‐EM population was characterized by expression of genes encoding activation markers, like *Tnfrsf4* (OX40) and *Cd40lg* (CD40 ligand), and *Ccr4* (CCR4), which has been associated with cutaneous recruitment [25]. After classifying Tcon into these populations, their relative abundance, quantified as a proportion of total nonneutrophil immune cells (CD45^+^Ly6G^−^), was tracked during *S. aureus* skin infection. As shown in Figure 2C, from day 13 p.i. onward, CD4‐EM emerged as the dominant Tcon population in murine skin. The accumulation of CD4^+^ T cells in the infected skin at Day 13 and Day 42 p.i. was confirmed using flow cytometry (Figure 2D).

Identical analyses were applied to γδ T cells, revealing three distinct subsets present within murine skin throughout *S. aureus* infection (Figure 2E,F). Distinct subsets were identified based on Vγ chain expression, with Vγ2 and Vγ4 cells being the major populations preinfection. From day 13 onwards, however, Vγ6Vδ4‐expressing cells represented the vast majority of γδ T cells within the skin (Figure 2G). FACS analysis confirmed the dominant γδ population present in the skin at Day 13 and 42 p.i. was negative for both Vγ1 and Vγ4 expression, a commonly used strategy to identify mouse Vγ6^+^ γδ T cells (Figure 2H). Unlike CD8^+^ T cells, CD4^+^ and γδ^+^ T cells were strongly elevated in the skin as a result of *S. aureus* infection, with CD4^+^ Trms being the most abundant T cell population at Day 42 p.i. (Figure 2I). Moreover, since the clonal expansion of Vγ6^+^Vδ4^+^ γδ T cells in the mouse skin following intradermal *S. aureus* infection has already been reported [26], we focused our attention on tissue‐resident CD4^+^ T cells.

### 3.3 *S. aureus* Skin Infection Results in the Formation of a Skin‐Resident Population of CD4^+^ Memory T Cells

**FIGURE 3:**
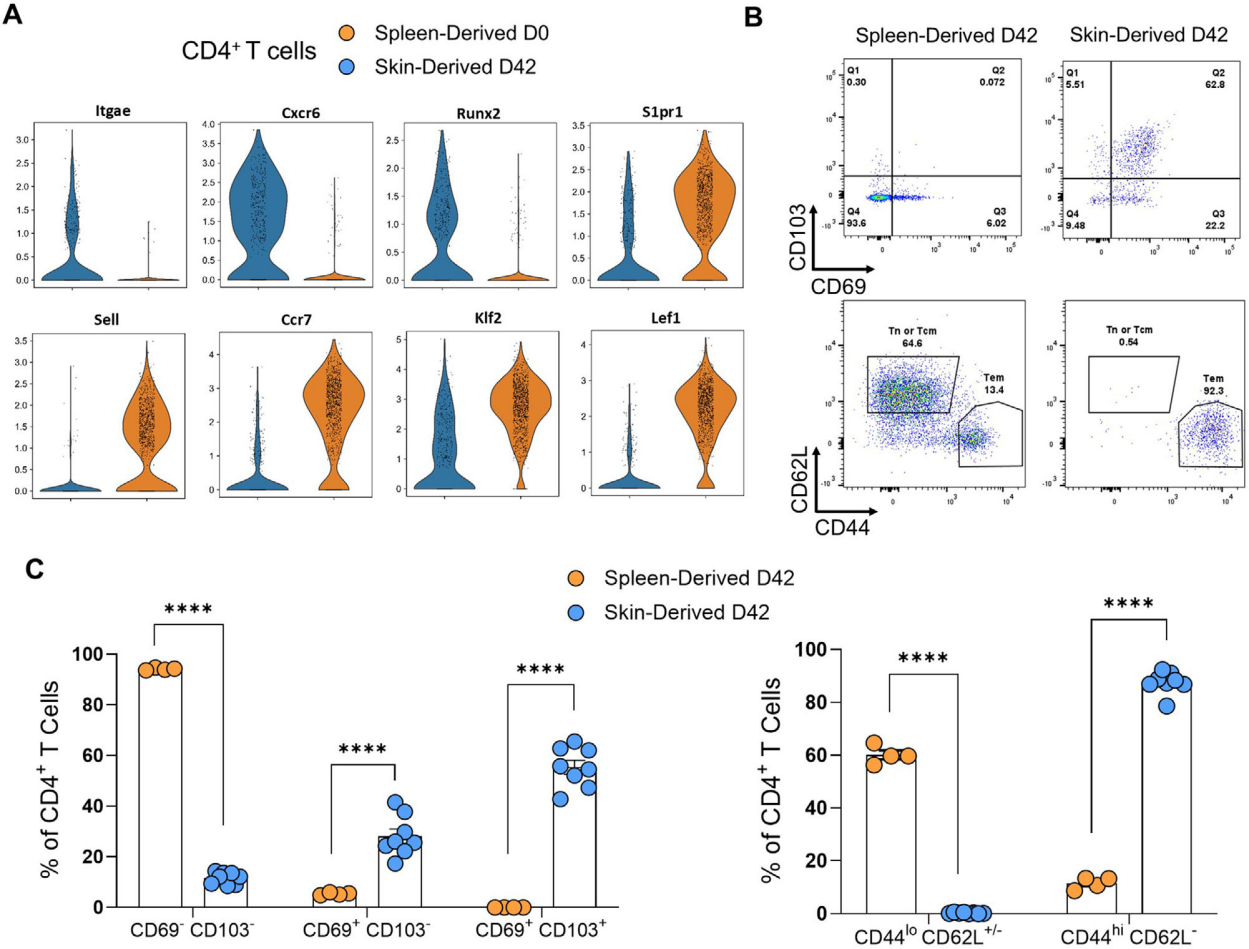
CD4^+^ T cells present in the skin display a tissue‐resident memory phenotype upon resolution of *S. aureus* infection. C57BL/6 female mice were infected subcutaneously with 2 × 10^7^ CFU of *S. aureus* USA300 LAC strain. The transcriptome of skin‐derived CD4^+^ conventional T cells (Tcon) isolated 42 days postinfection (p.i.) was compared against CD4^+^ Tcon cells from the spleen of naïve mice (*N* = 4/group). (A) ScRNA‐seq of FACS‐sorted single non‐neutrophil leukocytes (CD45^+^ Ly6G^−^) cells was performed, and the expression profile of genes related to tissue residency is reported. (B) Surface expression of the tissue‐residency markers CD69 and CD103 and the memory markers CD44 and CD62L were assessed on CD4^+^ T cells isolated from the skin (*N* = 8) and spleens (*N* = 4) of mice 42 days p.i. by FACS. Representative dot plots are shown. (C) Percentages of CD4^+^ T cells expressing these markers are shown. Statistical analysis was performed via two‐way ANOVA. ****p ≤ 0.0001.

While having previously been defined as CD4‐EM, the persistence and dominance of this CD4^+^ T cell population long after the resolution of infection raised the possibility that such cells may be Trms. To address this possibility, the transcriptional profile of total CD4^+^ Tcons at Day 42 p.i. was compared against CD4^+^ Tcons obtained from CD45^+^Ly6G^−^ splenocytes sorted and sequenced at baseline (Day 0). As splenocytes are associated with circulating immune responses, it was hypothesized that differentially expressed genes between splenic and skin‐derived CD4^+^ T cells may provide evidence of the adoption of tissue‐residency by skin‐derived CD4^+^ T cells at Day 42 p.i. Indeed, within the top 40 up‐ and downregulated genes between both groups (Figure S3), there was an abundance of genes associated with the adoption of a tissue‐residency profile in skin‐derived CD4^+^ T cells. The gene *Itgae* encoding CD103, known for its function in regulating T cell tissue‐residency [27], was upregulated in skin‐derived CD4^+^ T cells (Figure 3A). Commonly associated with CD8^+^ Trms, initial studies showed that the majority of CD4^+^ Trms in the genital mucosa were negative for CD103 expression [28]. Subsequent studies have shown, however, that CD103^+^ CD4^+^ Trms represent an appreciable Trm population in both humans and mice, albeit with tissue‐dependent variability [14, 18, 29]. The gene encoding the chemokine receptor CXCR6, which has been described as part of the transcriptional program of both CD4^+^ and CD8^+^ Trms [30, 31], was also upregulated in skin‐derived CD4^+^ T cells (Figure 3A). Similarly, the gene encoding the transcription factor RUNX2, which was upregulated in skin‐derived CD4^+^ T cells, is known to propagate skin‐residency in dermal CD8^+^ T cells (Figure 3A) [32]. Maintaining tissue‐residency is also associated with the downregulation of certain genes, particularly those related to tissue‐egress such as Sphingosine‐1‐phosphate receptor 1 (*S1pr1*), encoding an essential receptor mediating tissue egress into the circulation [33], or to lymphoid tissue homing such as *Sell* (encoding CD62L) and *Ccr7*. Indeed, we observed the downregulation of the transcription factor *Klf2*, known to enhance transcription of *S1pr1*, suggesting a layered transcriptional adaptation toward tissue residency [34]. The gene associated with stemness and T cell plasticity, *Lef1*, was downregulated in skin‐resident CD4^+^ T cells, further indicating their differentiation into a Trm phenotype [35].

To confirm that CD4^+^ T cells present in the skin 42 days p.i. were of a tissue‐resident memory phenotype, the expression of the tissue‐homing markers CD69 and CD103 and of the memory markers CD44 and CD62L was assessed via flow cytometry. Splenocytes were used as a control since it is expected that the majority of CD4^+^ T cells present in the spleen will not be tissue‐resident and should contain a mixture of memory phenotypes. Indeed, spleen‐derived CD4^+^ T cells were largely negative for the expression of CD69 and CD103, whereas the majority of skin‐derived CD4^+^ T cells were CD69^+^CD103^+^, with a smaller population being CD69^+^CD103^−^ (Figure 3B, upper dot‐plots). When compared with splenocytes from the same infected mice, a significant increase in the proportion of these populations in the skin‐derived CD4^+^ T cells was observed too (Figure 3C).

CD44 and CD62L can be used to distinguish naïve (Tn, CD44^lo^CD62L^+^), central memory (Tcm, CD44^hi^CD62L^+^) and effector memory (Tem, CD44^hi^CD62L^−^) populations [36, 37]. While spleen‐derived CD4^+^ T cells contained all these populations, skin‐derived CD4^+^ T cells were composed of a homogeneous Tem population at Day 42 p.i. (Figure 3B, lower dot‐plots). Indeed, when compared against splenocytes from the same mice, a significant increase in Tem was observed in the skin (Figure 3C). Taken together, these results demonstrate that skin infection with *S. aureus* results in the development of a skin‐resident memory population of CD4^+^ T cells that persists after resolution of infection.

### 3.4 Skin‐resident Memory CD4^+^ T Cells Display Elevated Effector Functions and Could Contribute to Protective Immunity During Reinfection With *S. aureus*

**FIGURE 4:**
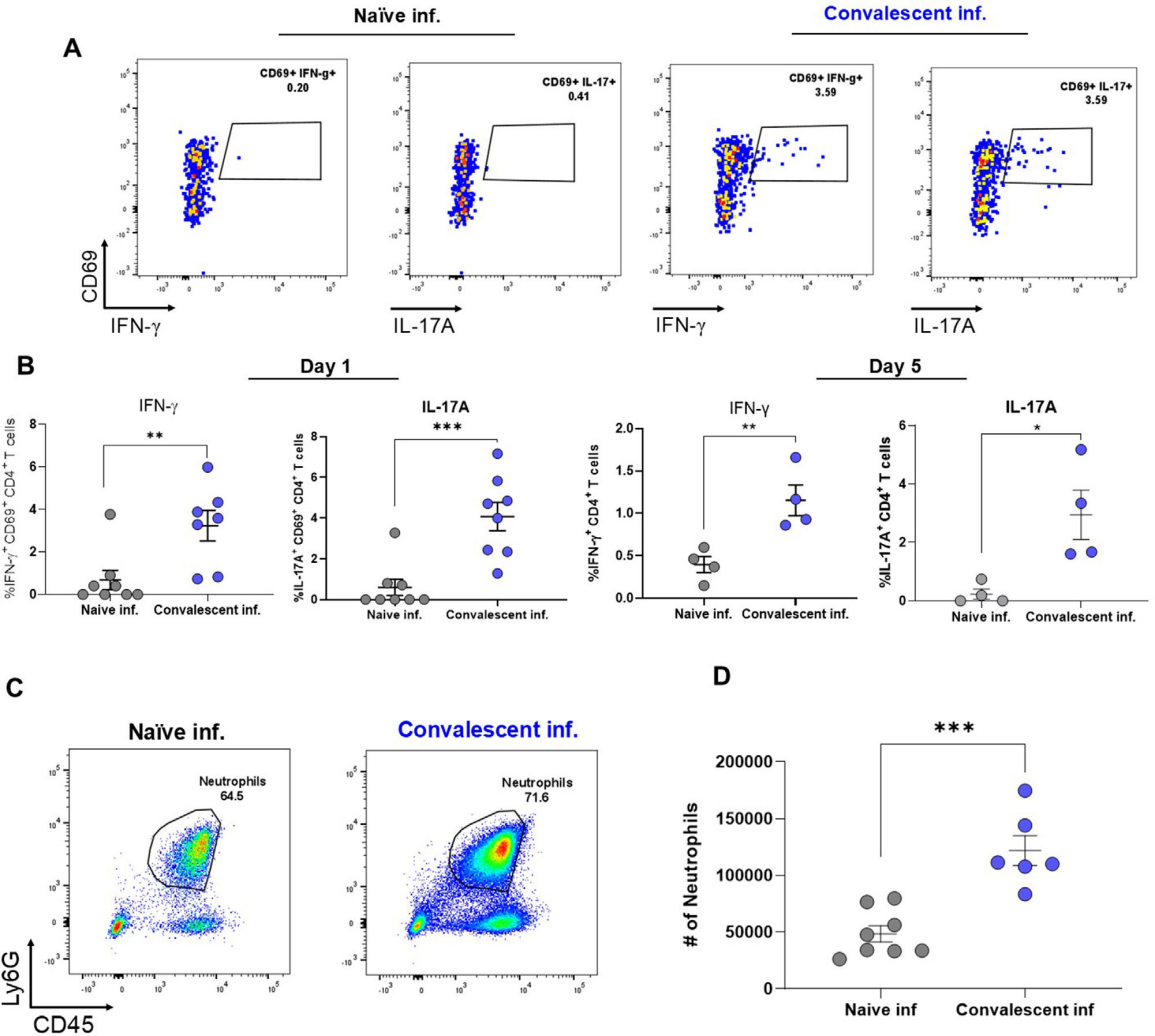
Skin‐resident CD4^+^ T cells display enhanced immune responsiveness during secondary *S. aureus* skin infection. C57BL/6 female mice were infected subcutaneously with 2 × 10^7^ CFU of *S. aureus* USA300 LAC strain or treated with PBS. After 42 days, both groups were infected with *S. aureus* (convalescent inf. or naïve inf., respectively), and the production of the pro‐inflammatory cytokines IFN‐γ and IL‐17A was measured in skin‐derived CD4^+^ T cells 24 h and 5 days post infection (p.i.) by intracellular cytokine staining. (A) Representative flow plots showing the production of both cytokines in combination with CD69 expression. (B) The percentage of CD4^+^ T cells producing each cytokine at 24 h or on day 5 p.i. was quantified in individual mice. (C) The infiltration of neutrophils (CD45^+^Ly6G^+^) in the skin of naïve infected or convalescent infected mice was measured 24 h p.i.; representative flow plots gated on live, single cells are shown. (D) The number of neutrophils present in infected skin from individual mice was quantified. Statistical analysis was performed via Student's *t*‐test. Results are displayed as mean ± SEM. *p ≤ 0.05. **p ≤ 0.01. ***p ≤ 0.001.

To understand whether CD4^+^ Trms induced by a first *S. aureus* skin infection become reactivated during a second infection, mice were infected as described previously and allowed to recover for 42 days, at which point convalescent mice were rechallenged with *S. aureus* in the same way as before. IL‐17 and IFN‐γ production by skin‐derived CD4^+^ T cells was assessed *ex vivo* with co‐stimulation using α‐CD28 antibodies and subsequent intracellular cytokine staining 1 and 5 days post‐rechallenge with *S. aureus*. For comparison, naïve mice injected with PBS on day 0 and infected with *S. aureus* on day 42 were analyzed in parallel. Significantly increased percentages of skin‐derived CD4^+^ T cells producing both cytokines were observed in convalescent infected mice at each timepoint (Figure 4A,B). Consistent with the increase in IL‐17 production observed 1 day post reinfection, a significant numerical increase in skin‐infiltrating neutrophils was recorded in convalescent compared with naïve infected mice at this timepoint (Figure 4C,D). γδ T cells were also investigated for IL‐17 and IFN‐γ production at the corresponding time points. While numerically there were increased numbers of γδ T cells during secondary infection, there was only a marginal increase in the percentage of γδ T cells that produced either IL‐17 or IFN‐γ (Figure S4).

**FIGURE 5:**
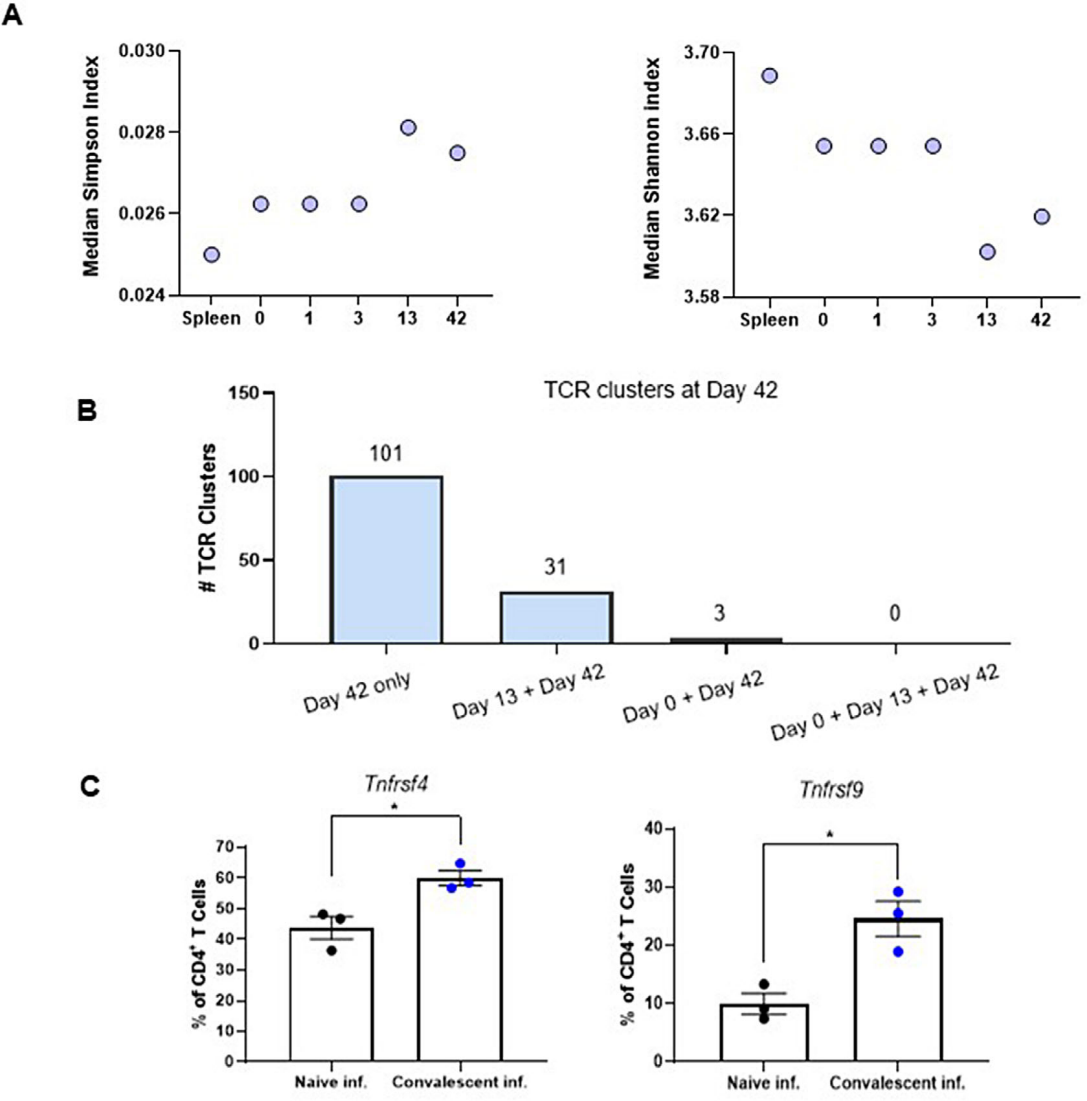
TCR sequencing data support *S. aureus* specificity of CD4^+^ Trm cells present in the skin after first *S. aureus* infection. (A) C57BL/6 female mice were infected subcutaneously with 2 × 10^7^ CFU of *S. aureus* USA300 LAC strain. At days 0 (non infected), 1, 3, 13, and 42 post infection (p.i.), skin was harvested, a single‐cell suspension was prepared, and stained. Live, single non‐neutrophil leukocytes (CD45^+^ Ly6G^−^) were FACS‐sorted, TCR‐seq was performed, and data were analyzed. Simpson and Shannon indices of TCRs sequenced across skin samples taken at different times were calculated. Higher Simpson index and lower Shannon index values were observed at Day 13 and 42 p.i. compared with Day 0, indicating a higher clonal expansion and a lower diversity in TCRs at these time points after infection. For comparison, these indices were also calculated for non infected spleen samples. (B) Clonotype clustering using TCRdist3 was used to identify TCR clusters in the CD4^+^ Trm present in the skin of mice on day 42 p.i. The presence of the 135 clonotypes identified at this time point at Day 13 p.i. and/or at Day 0 (no infection) was analyzed. The numbers of clonotypes found at Day 42 p.i. only or also at Day 13 are reported. (C) The percentages of CD4^+^ T cells transcribing the genes *Tnfrsf4* and *Tnfrsf9* encoding the activation markers OX40 and CD137, respectively, present in the skin 24 h after primary or secondary *S. aureus* skin infection were measured by scRNA‐seq. Statistical analysis was performed via Student's *t*‐test. Results are displayed as mean ± SEM. *p ≤ 0.05.

To understand the clonality of the CD4^+^ Trm response, we performed single‐cell TCR sequencing. Trends toward increases and decreases in the Simpson and Shannon indices, respectively, were observed throughout the time course of infection, indicating potential expansion of antigen‐specific clonotypes during the course of infection (Figure 5A). Using TCRdist3‐based clonotype clustering on our cross‐sectional TCR‐seq data, we identified 135 TCR clusters in the CD4^+^ Trm present in the skin of mice at Day 42 p.i. Remarkably, 31 of these TCR clusters were also found at Day 13, compared with only 3 clusters shared between Day 42 p.i. and Day 0. The TCR cluster overlap between Day 13 (acute Teff) and Day 42 p.i. (established Trm) suggests that the CD4^+^ Trm that are present in the skin of convalescent mice may derive from Teff that infiltrated during primary infection (Figure 5B). The transcription of *Tnfrsf4* and *Tnfrsf9* genes, encoding the activation markers OX40 and CD137, respectively, was elevated 24 h after secondary infection compared with that 24 h after ndergoing a primary infection 24 h p.i. (Figure 5C). Given that these markers are commonly used in activation‐induced marker assays due to their being refractory to the influence of nonspecific activation by cytokines [38, 39]. These data suggest that the skin‐resident CD4^+^ Trm response is at least partially specific for *S. aureus*.

**FIGURE 6:**
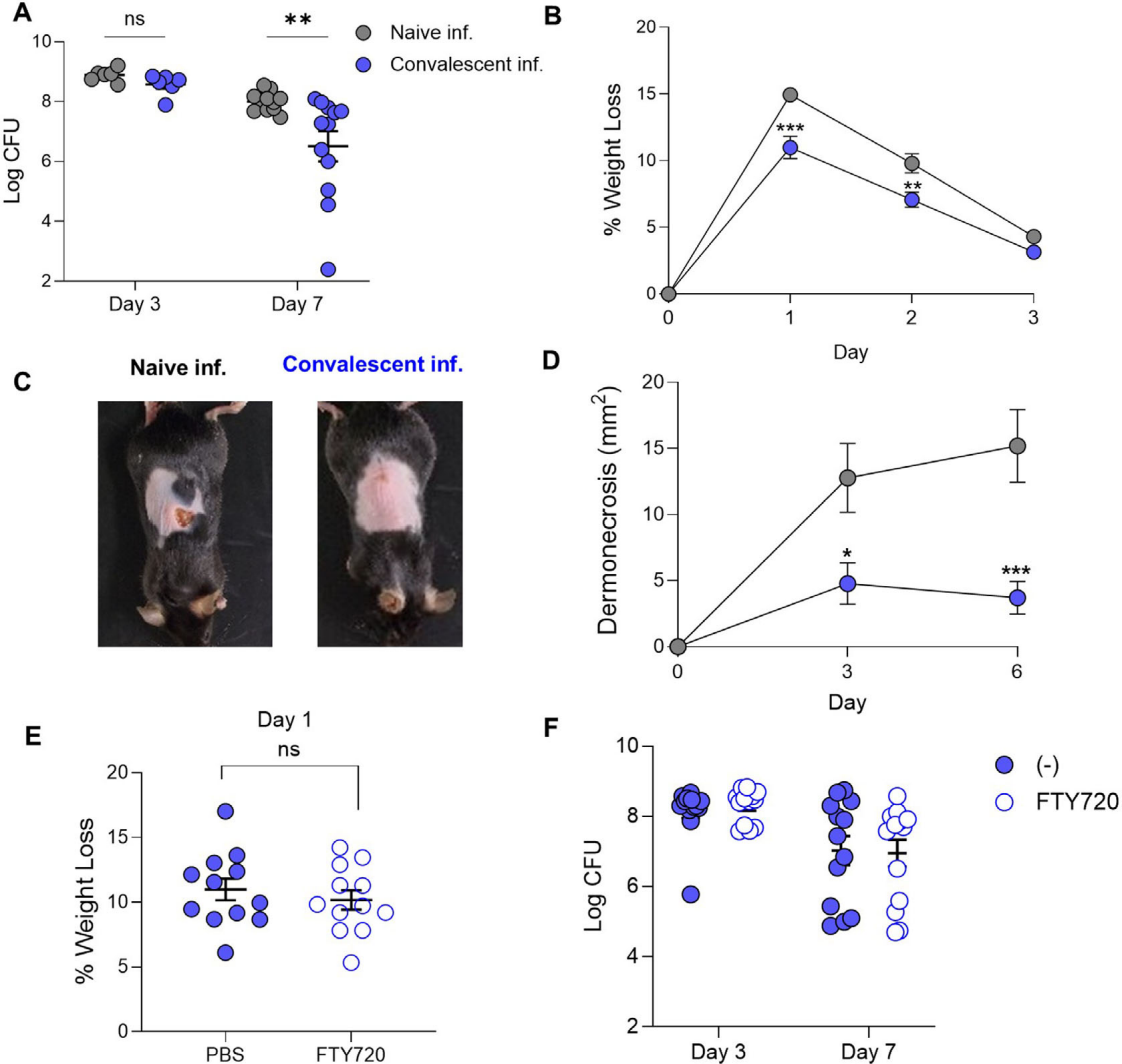
First skin infection with *S. aureus* partially protects from reinfection independently of lymphocyte egress from secondary lymphoid organs. C57BL/6 female mice were infected subcutaneously with 2 × 10^7^ CFU of *S. aureus* USA300 LAC strain or treated with PBS. After 42 days, both groups were infected with *S. aureus* (convalescent inf. and naïve inf., respectively). (A) After 3 or 7 days, CFU present in the skin were enumerated. (B) On days 1, 2, and 3 post infection (p.i.), the body weight of individual mice was monitored. For each mouse, loss of body weight was calculated as a percentage of that immediately before infection. The development of dermonecrosis was monitored at days 3 and 6 post‐rechallenge. The area of formed dermonecrosis was measured via photography using an object of defined length for scale reference. (C) Representative images taken at day 3 p.i. of naïve inf. and convalescent inf. mice are shown. (D) Dermonecrosis at separate time points is presented. (E, F) Convalescent mice were treated with FTY720 immediately before and throughout reinfection or with PBS as a control. (E) 24 h p.i., bodyweight was measured, and weight loss was compared between the two groups. (F) On day 3 and 7 p.i., infected skin was harvested, and CFU were enumerated (*N* = 12–13 mice/group, two independent experiments). Statistical analysis was performed via two‐way ANOVA (A, B, D) or *t*‐test (E). *p ≤ 0.05. **p ≤ 0.01. ***p ≤ 0.001.

These data suggest that skin‐resident memory T cells induced by an initial infection may contribute to a protective immune response through localized, enhanced cytokine expression. Importantly, convalescent mice had reduced bacterial burdens in the skin as compared with naïve mice 7 days p.i. (Figure 6A). In addition, convalescent mice displayed a significant reduction in weight loss both 1 and 2 days p.i. that returned to normal by day 3 p.i. in both groups, reflecting the acuteness of this infection model (Figure 6B). Convalescent mice developed less severe dermonecrosis during reinfection when compared with naïve infected mice (Figure 6C,D). Taken together, these results demonstrate that mice develop partially protective immunity during recurrent *S. aureus* skin infection, which is associated with an accumulation of IL‐17 and IFN‐γ‐producing Trms within the skin, which our group had previously hypothesized [22].

To test whether blocking the infiltration of lymphocytes into skin would affect protection against *S. aureus* reinfection, convalescent mice were treated with FTY720, which, by inducing S1P receptor internalization and degradation on lymphocytes, prevents their egress from secondary lymphoid organs to the circulation. Despite the depletion of skin‐infiltrating lymphocytes, no differences were observed between FTY720‐ and PBS‐treated mice p.i. in terms of weight loss after 24 h (Figure 6E) or bacterial burdens at days 3 and 7 post‐reinfection (Figure 6F). These results are compatible with a protective role of skin‐resident memory T cells induced by the first *S. aureus* infection toward reinfection.

## 4 Discussion

A critical bottleneck in *S. aureus* vaccine development and past failures is the lack of defined correlates of protection [40]. Perturbations to CD4^+^ T responses are known to predispose individuals to the development of *S. aureus* infections, particularly SSTIs. Furthermore, it has been demonstrated that different vaccine compositions may be required for tissue‐related versus systemic infections with *S. aureus* [41]. As a result, it was hypothesized that CD4^+^ Trms may serve as a previously unidentified correlate of protection during *S. aureus* SSTI [22]. Despite this, to the best of our knowledge, a thorough investigation into Trm development during *S. aureus* skin infection is lacking. In this study, a deep and high‐resolution characterization of the cellular immune response during *S. aureus* skin infection was described. A population of CD4^+^ Trm was identified that seeded the skin in response to initial infection with *S. aureus*. This population of Trms responded quickly and vigorously to re‐challenge with *S. aureus*, producing IL‐17 and IFN‐γ. Furthermore, it was shown that protective immunity was independent of infiltrating lymphocytes, suggesting that CD4^+^ Trm could contribute to *S. aureus* immunity. Therefore, targeting *S. aureus*‐specific Trms through vaccination might be a way to improve vaccine efficacy.

An established acute model of *S. aureus* skin infection using the USA300 LAC strain was used in this study. In early time points p.i. (Day 1 and 3) there was a massive infiltration of neutrophils and monocyte/macrophages, followed by NK cells and, surprisingly, B cells (Day 3), which displayed less of an adaptive‐like kinetic than T cell populations that infiltrated the skin at Day 13 and remained high at Day 42 when bacteria were cleared and skin was repaired. In particular, a CD4^+^ T effector memory population, subsequently defined as Trm, was the dominant population at Day 42 p.i. In addition, γδ T cells were found to be enriched in mouse skin post infection. Replacement of initial Vγ2 and Vγ4 populations with a Vγ6 population echoes previous studies in which this subset was shown to expand upon *S. aureus* or *Listeria monocytogenes* infection and execute memory responses upon reinfection [26, 42, 43, 44]. To our knowledge, this study shows, for the first time, that a memory population of γδ T cells can form within the skin tissue itself, with future investigations into their immunological potential highly warranted.

Interestingly, transcriptional analysis revealed multiple genes in CD4^+^ Trms typically associated with CD8^+^ Trm formation and maintenance, such as the downregulation of *Klf2* or upregulation of *Itgae* and *Runx2* [27]. Considering CD8^+^ Trms have to date been studied in greater detail than their CD4^+^ counterparts, it is likely that similar transcriptional programs may be at work to maintain tissue residency in both subsets, as the data presented here suggest. While a proportion of CD4^+^ Trms found in the skin of infected mice at Day 42 p.i. were CD69^+^CD103^−^, the majority of cells were found to be double positive for expression of these Trm markers. Remarkably, these CD4^+^ Trms readily produced IL‐17A and IFN‐γ during secondary infection with *S. aureus* compared with naïve mice undergoing primary infection, which further correlated to increased neutrophil recruitment during secondary infection. While we did not causally link this enhancement of cytokine production to neutrophil recruitment, it is well established that IL‐17 is crucial for the recruitment of neutrophils to tissues and the concomitant clearance of microbes such as *S. aureus* during infection [45, 46, 47, 48, 49]. Furthermore, protective immunity induced by initial infection was unperturbed by FTY720 treatment, suggesting that skin‐resident CD4^+^ Trms could mediate immunological memory. As FTY720 does not discriminate between lymphocyte populations, it is possible that γδ T cells are also contributing toward a protective role in conjunction with CD4^+^ T cells. It is also important to note that FTY720 will not inhibit antibody responses, which have been shown in many models of *S. aureus* infection and vaccination to contribute to protective immunity [7, 50, 51, 52, 53]. The strongest and most consistent antibody‐based correlate of reduced risk and severity of *S. aureus* SSTIs is functional neutralizing activity against α‐toxin (Hla), supported by convergent extensive mechanistic evidence mainly from murine skin‐infection models [7, 54] and human data on recurrence risk in children and infants [55, 56], but not in older subjects. Remarkably, human Trms undergo site‐specific development and progressive maturation over infancy and childhood to reach a similar frequency, phenotype, and function as adult Trms across sites by the age of 4–5 years [57]. We propose that this could be the reason why the protective role of antibodies becomes less relevant from this time on.

In agreement with our findings, a study by Braun et al. demonstrated that atopic dermatitis flares induced by repeated topical applications of *S. aureus* strains isolated from AD patients were unperturbed when FTY720 was administered during a memory timepoint, linking inflammation to skin‐resident CD4^+^ and γδ T cells [44]. Importantly, the aforementioned study also reported that these skin‐resident memory T cells reduced the bacterial load in the skin. Therefore, we propose that such a response could be harnessed via vaccination. Indeed, a growing body of literature suggests that Trms are key mediators of protection in different murine models of *S. aureus* infection. Braverman and colleagues demonstrated that lung‐resident CD4^+^ T cells induced by an *S. aureus* antigen‐expressing influenza virus infection can limit the severity of a subsequent *S. aureus* infection [21]. Taken together, mounting evidence reinforces the potential for Trms to act as critical mediators of protective immunity during *S. aureus* infection that could be targeted, in conjunction with a humoral response, through vaccination.

Applying such a vaccination strategy to the skin must account for the fact that CD4^+^ Trms specific to *S. aureus* are already present within the skin of healthy individuals [23]. In the present study, expanding a clonal population was associated with the development of protective immunity. Whether or not pre‐existing human CD4^+^ Trms are protective in the context of infection will be essential to delineate for future vaccination strategies. In a scenario of pre‐existing Trms proving protective, inducing their expansion may prove beneficial. This can be achieved through localized boosting of Trms as shown in murine studies via the topical addition of antigen to the skin surface [58, 59, 60]. Inducing a de novo skin‐focused immune response could be achieved through intradermal vaccination, which conveys the added benefit of being highly immunogenic in inducing systemic immune responses as well [61, 62]. Work from this group recently showcased that circulating *S. aureus*‐specific T cells include a population of TIGIT^+^ Treg that will likely need to be overcome for the optimal immunogenicity of any future *S. aureus* vaccine [63].

In summary, this work provides a high‐resolution immunological profile of the kinetics of *S. aureus* skin infection. Doing so, γδ and particularly CD4^+^ T cells emerged as key populations of tissue‐resident memory T cells that persist after infection, with CD4^+^ Trms being shown to respond more vigorously to re‐infection with *S. aureus*. Combined with a growing body of recently published literature, the data presented here underscore the exciting potential of Trms as candidates in the search for a vaccine‐inducible immune response to combat *S. aureus* infections.

## Author Contributions

J.C.: Conceptualization, data curation, formal analysis, Writing – original draft, review and editing. G.C.: Conceptualization, supervision, data curation, formal analysis. A.C.: Data curation, formal analysis, methodology. M.S.: Data curation, Formal analysis, Project administration. E.C.: Conceptualization, supervision, project administration. S.T.: Data curation, methodology. CS: Data curation, methodology. N.R.: Data curation, formal analysis, methodology. S.V.: Data curation. S.G.: Data curation, methodology. E.Si. and M.Ba.: Conceptualization, supervision. F.B.: Conceptualization, supervision, project administration, resources. M.Br.: Conceptualization, supervision, funding acquisition, project administration. R.McL.: Conceptualization, supervision, funding acquisition, project administration, Writing – review and editing. E.So.: Conceptualization, supervision, funding acquisition, project administration, resources, Writing – review and editing.

## Funding

Associated research in the McLoughlin lab was funded by a Research Ireland Frontiers for the Future Award (23/FFP‐A/12244) and a Wellcome Discovery Award (338537/Z/25/Z) to R.M.M.

## Conflicts of Interest

Giovanni Cova, Simona Tavarini, Chiara Sammicheli, Nicholas Rachmaninoff, Emiliano Chiarot, Emilio Siena, Monia Bardelli, Michela Brazzoli, Fabio Bagnoli, and Elisabetta Soldaini are employees of the GSK group of companies. Jonah Clegg, Megan Smith, and Serena Vastola were PhD students at GSK during the study. Alberto Carignano and Giovanni Cova were part of the Scientific Leadership Program at GSK Vaccines during the study. Fabio Bagnoli is listed as an inventor on patents of *S. aureus* vaccine candidates owned by GSK. Emilio Siena, Monia Bardelli, Michela Brazzoli, Emiliano Chiarot, Fabio Bagnoli, and Elisabetta Soldaini report ownership of GSK shares and/or restricted GSK shares. The remaining authors declare no conflicts of interest.

## Declaration of Generative AI and AI‐Assisted Technologies in the Writing Process

During the preparation of this work, the authors used ChatGPT in parts of the manuscript to improve the readability and language of the manuscript. After using this tool, the authors reviewed and edited the content as needed and take full responsibility for the content of the publication.

## Supporting information




**Supporting File**: eji70268‐sup‐0001‐SuppMat.docx.

## Data Availability

All sequencing data have been prepared to be submitted in an open‐access online repository, including coding notebooks demonstrating analysis. Other data included within the paper are available upon request.
