# Supplementary material for: Single‐Cell Profiling Identifies Skin‐Resident Memory CD4+ T Cells as a Potential Correlate of Immunity During Staphylococcus aureus Skin Infection
Source: Eur J Immunol. 2026 Aug 24;56(8):e70268. doi: 10.1002/eji.70268 (PMC13501939; doi:10.1002/eji.70268)
Supplement: Supplementary file 1 [file EJI-56-e70268-s001.docx]

**Supplemental Figure 1.** S. aureus skin infection is self-resolving and involves significant neutrophil infiltration.

(A-B) C57BL/6 female mice were injected subcutaneously with *S. aureus* (2 x 10^7^ CFU) at day 0. Skin was harvested at varying timepoints, homogenised and then bacterial CFU were enumerated by plating on TSA agar (N = 5 individual mice/timepoint) (B). The presence of neutrophils was measured via flow cytometry by gating on live single CD45^+^Ly6G^+^ cells. Representative FACS plots are shown (C). Neutrophils as a percentage of total CD45^+^ immune cells were quantified (N = 3-10 individual mice/timepoint) (D). Statistical analysis was performed comparing each timepoint against day 0 (no infection) using a one-way ANOVA with Dunnet’s test for multiple comparisons. **** P≤0.0001.

**Supplemental Figure 2.** The top 5 cluster-defining genes of each immune cell subset.

The top 5 differentially expressed genes in each of the 14 distinct immune populations identified via Leiden clustering shown in Fig. 1A are shown. For aesthetic reasons, the table was split into 2 parts. Dot size relates to the fraction of cells in each population actively transcribing a given gene whereas colour intensity relates to the mean, relative expression level of a given gene for each population (N = 4 mice for each timepoint).

**Supplemental Figure 3.** Differentially expressed genes between splenic and skin-resident CD4^+^ T cells

C57BL/6 female mice were infected subcutaneously with 2 x 10^7^ CFU of *S. aureus* strain USA300. The transcriptome of skin-derived CD4^+^ conventional (Tcon) cells isolated 42 days post-infection was compared against CD4^+^ Tcon cells from the spleen of naïve mice (N = 4). ScRNA-seq was performed and the top 20 differentially expressed genes are reported.

**Supplemental Figure 4** γδ T cell response during secondary *S. aureus* infection

C57BL/6 female mice were infected subcutaneously with 2 x 10^7^ CFU of *S. aureus* USA300 or treated with PBS. After 42 days, both groups were infected with *S. aureus* (convalescent inf. or naïve inf., respectively) and the production of the pro-inflammatory cytokines IFN-γ and IL-17A was measured in skin-derived γδ T cells 24 h and 5 days post infection (p.i.) by intracellular cytokine staining. (A) Quantification of γδ T cells in the skin 24 h p.i. (B) Representative flow plots showing the production of both cytokines in combination with CD69 expression. (C) The percentage of γδ T cells producing each cytokine at 24 h or on day 5 p.i was quantified in individual mice. (C, D) Statistical analysis was performed via student’s t-test. Results are displayed as mean ± SEM. **P≤0.01.
